# The complete mitochondrial genome of *Lasiopodomys brandtii*

**DOI:** 10.1080/23802359.2019.1703567

**Published:** 2020-01-07

**Authors:** Xiangyu Tian, Shiming Gu, Dan Pan, Luye Shi, Zhenlong Wang

**Affiliations:** School of Life Sciences, Zhengzhou University, Henan, Zhengzhou, P. R. China

**Keywords:** Complete mitochondrial genome, *Lasiopodomys brandtii*, phylogenetic tree

## Abstract

In this study, the complete mitochondrial (mt) genome sequence of *Lasiopodomys brandtii* was determined using Illumina NovaSeq platform. The assembled genome was 16,557 bp in length and included 13 protein-coding genes, 2 ribosomal RNA genes, and 22 transfer RNA genes. The total nucleotide composition frequencies present clearly the A-T skew (59.5%), which mostly in D-loop and PCGs regions. Whole mt genome phylogenetic analysis revealed a closely related among *Lasiopodomys*, *Proedromys*, and *Microtus* with high support. It would provide further evolutionary research for the subfamily Arvicolinae.

Brandt’s vole (*Lasiopodomys brandtii*), known as the colonial rodent species of the typical temperate grassland, which is distributed Inner Mongolia of China, east of the Republic of Mongolia, southern of Russia (Zhang et al. 2003; Alexeeva et al. 2015). Brandt’s vole is widespread living above ground in a diverse habitats (Dong et al. 2018), as the main food resource of most predators played an important role in grassland ecosystem. The species *L. brandtii* with *Lasiopodomys fuscus* and *Lasiopodomys mandarinus* shared the similar morphologically and merged into the genus *Lasiopodomys* from a subgenus of *Microtus*, all of them are belong the subfamily Arvicolinae (Wilson and Reeder 2005). Some studies suggested that *L. fuscus* was not closely with *L. brandtii*, should be removed to *Neodon* (*Neodon fuscus*) based on molecular phylogenetic (Abramson et al. 2009; Bannikova et al. 2010; Liu et al. 2017). To date, only COI and cytb mitochondrion sequences submitted to GenBank, there is no study on the whole mitochondrial genome of this species.

In this study, *L. brandtii* specimen was stored at Herbarium of School of Life Sciences, Zhengzhou University (specimen no.: LZ002), which was collected from the field of Huairou, Beijing, China (40.896°N, 116.636°E). The total genomic DNA was extracted from the *L. brandtii* muscle tissue using TIANamp Genomic DNA Extraction Kit (TIANGEN, Beijing, China; DP304), sequenced using Illumina NovaSeq 6000 (Illumina Co., San Diego, CA, USA), assembled using NOVOPlasty version 3.6 (Dierckxsens et al. 2016). The *L. brandtii* mitochondrial (mt) genome is 16,557 bp (GenBank accession number: MN614478), contains 13 protein-coding genes (PCGs), 22 tRNA genes, 2 rRNA genes, and a control region (D-loop). Among these genes, All PCGs began with ATN codons (ND4, COX3 starts with ATC, ATT), except for ND1 with GTG as the alternative initiation codons. The typical stop codon TAA has been assigned to 10 PCGs, BESIDES, ND1, and ND6 are terminated with TAG, COX3 has TAC stop codon. The total nucleotide composition frequencies of *L. brandtii* mt genome were A (32.1%), T (27.4%), C (26.4%), and G (14.0%), clearly emerge the A/T skew, which was generally apparently in the D-loop and PCGs.

In order to investigate the phylogenetic position of *L. brandtii*, we used complete mitochondrial genome sequence include eight related species (two sequences of *L. mandarinus*) and *Cricetulus kamensis* as the outgroup derived from NCBI. The phylogenetic analysis was used BEAST version 1.8.3 software to construct a Bayesian inference with 50,000,000 generations using default parameters (Drummond et al. 2012). Results of phylogenetic tree showed *L. brandtii* and *L. mandarinus* grouped into a single clade (Figure 1). Both of them had a close relationship with genus *Proedromys* and *Microtus* (BP = 100). In summary, this study confirms the mt genome sequence would be an important marker for the *Lasiopodomys* and related genus evolutionary.

**Figure 1.**
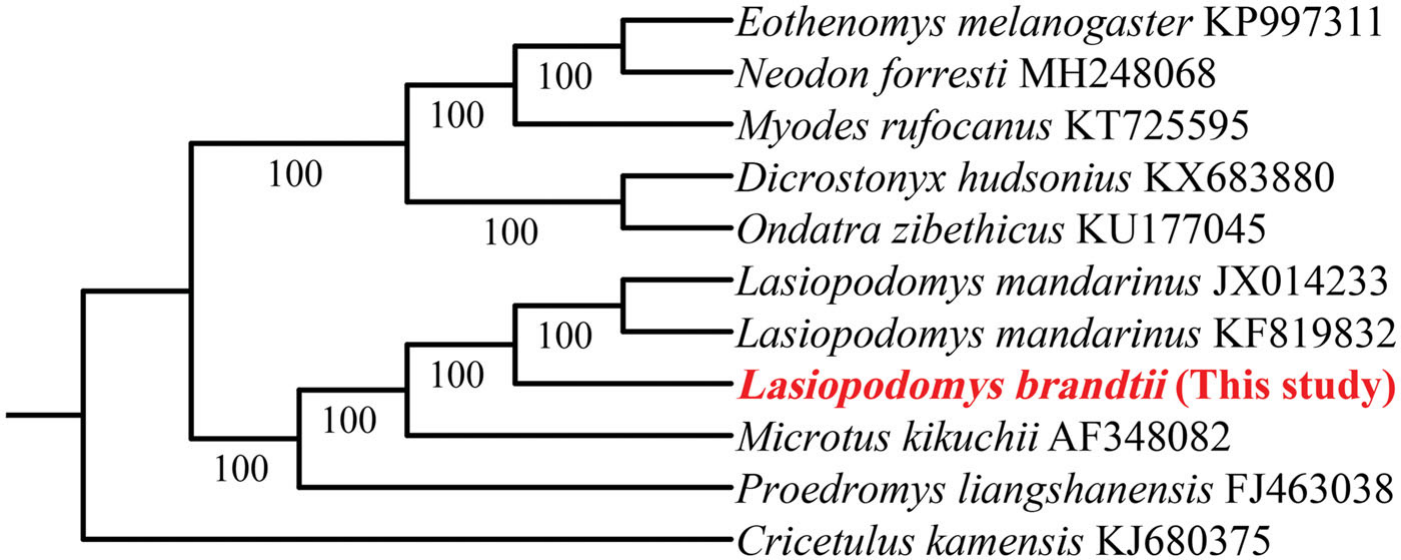
Bayesian inference phylogenetic tree of *Lasiopodomys brandtii* and eight other species using *Cricetulus kamensis* as the out group. Number below each branch indicates posterior probabilities support values.
